# Can we avoid intracranial complications of chronic otitis media?

**DOI:** 10.1007/s00405-014-3411-x

**Published:** 2014-12-04

**Authors:** Jerzy Kuczkowski, Dmitry Tretiakow, Wojciech Brzoznowski

**Affiliations:** 1 Department of Otolaryngology, Medical University of Gdansk, Gdansk, Poland; 2 ENT Department, Medical University of Gdansk, Dębinki 7, 80-952 Gdansk, Poland

We have read with great pleasure the article written by Jiaqiang Sun and Jingwu Sun [1] on the problem of intracranial complications (IC) of chronic otitis media (COM). It is a useful publication reminding us of the dangers of IC of COM. Has anything changed in the clinical picture of these complications? In the author’s material, the most common IC were brain abscess (52.9 %) and isolated bacterial colonies were *Proteus mirabilis*, anaerobes, *Staphylococcus aureus* and *Pseudomonas aeruginosa* [1]. However, misuse or overuse of antibiotics evoked development of drug resistance of certain bacterial strains causing chronic otitis media.

The middle ear colonization by anaerobic flora, *Staphylococcus aureus and Pseudomonas aeruginosa* is the cause of recurrent exacerbations of COM and as a consequence IC. At least 65 % of human infectious diseases are caused by biofilms which are produced by many microbes. According to Xingzhi Gu et al. [2], bacterial biofilms were present in 85–92 % of patients with COM. Difficult ear drainage (anatomic structure abnormalities, mucosal edema, polyps, cholesteatoma, exostoses) is a direct consequence of COM. Inflammatory response to bacterial exudates (anaerobes or *Pseudomonas aeruginosa*) in the tegmen tympani or cochlea causes bone destruction or vein thrombosis which can lead to IC [2–4].

**Fig. 1:**
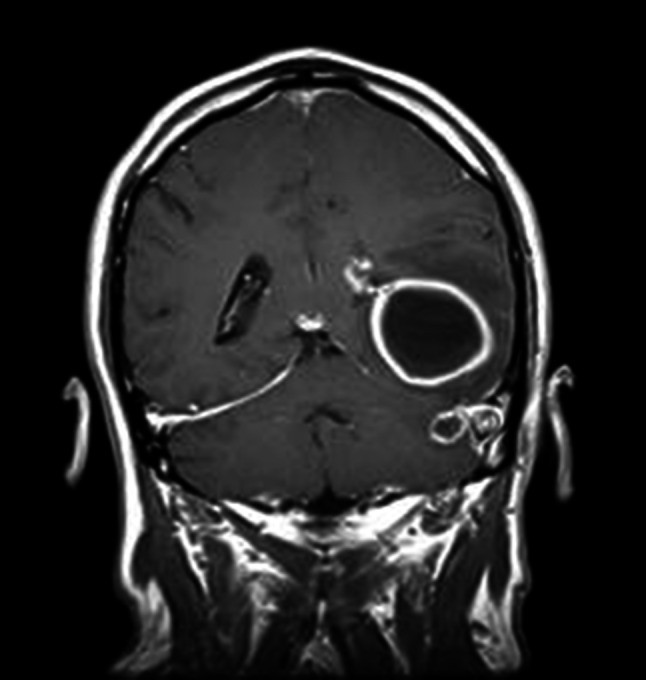
MRI image of the head in coronal plane shows the circular pathological mass in the left temporal lobe-abscess (the 52-year-old patient)

Exacerbation of the middle ear infection may be the indication for the use of an antibiotic which may mask, delay or suppress the symptoms of IC, allowing the diagnosis only when neurological symptoms are visible. Antibiotic therapy without ear drainage may also contribute to development of IC. Mechanical removal of biofilms may allow to improve treatment efficacy in COM. The six patients with chronic otitis media with cholesteatoma and IC were treated in our department between 2006 and 2012. *Pseudomonas aeruginosa* and *Staphylococcus aureus* were the most common isolated bacteria. Diagnostic tools such as CT or MRI and urgent surgical intervention allowed to treat all patients without neurological deficiencies. In the case of significant bone destruction, we used open technique in all cases. Based on the authors’ article and our experience and observations, we can assume that the etiological factors responsible for IC are biofilms produced by bacteria. Presence of a biofilm in the ear can contribute to COM exacerbation and as a result, bone destruction and IC. Clinical picture of COM has slightly changed in comparison to those from previous decades. Symptoms are at present less obvious and severe. Failure to improve the condition of patients after antibiotic treatment suggests that we have encountered with “resistant” biofilms. At the present time it is difficult to avoid IC development. The thorough ear toilet, irrigation and applying drugs destroying the structure of the biofilms may be an important way to avoid IC. The latest assumption requires forward studies, because we do not have at our disposal the effective drugs destroying biofilms (Fig. 1).
